# Competitive helical bands and highly efficient diode effect in F/S/TI/S/F hybrid structures

**DOI:** 10.3762/bjnano.17.2

**Published:** 2026-01-05

**Authors:** Tairzhan Karabassov, Irina V Bobkova, Pavel M Marychev, Vasiliy S Stolyarov, Vyacheslav M Silkin, Andrey S Vasenko

**Affiliations:** 1 HSE University, 101000 Moscow, Russia; 2 Moscow Institute of Physics and Technology, Dolgoprudny, Moscow 141700, Russia; 3 Dukhov Research Institute of Automatics (VNIIA), 127055 Moscow, Russia; 4 Donostia International Physics Center (DIPC), San Sebastián/Donostia, 20018 Basque Country, Spain; 5 Departamento de Física de Materiales, Facultad de Ciencias Químicas, UPV/EHU, 20080 San Sebastián, Basque Country, Spain; 6 IKERBASQUE, Basque Foundation for Science, 48011 Bilbao, Spain; 7 Department of Physics, Moscow State University, Moscow 119992, Russia

**Keywords:** hybrid structures, proximity effect, superconducting diode effect, superconductivity, topological insulators

## Abstract

The diode effect in superconducting materials has been actively investigated in recent years. Plenty of different devices have been proposed as a platform to observe the superconducting diode effect. In this work, we discuss the possibility of a highly efficient superconducting diode design with controllable polarity. We propose a mesoscopic device that consists of two separated superconducting islands with proximity-induced ferromagnetism deposited on top of a three-dimensional topological insulator. Using the quasiclassical formalism of the Usadel equations, we demonstrate that the sign of the diode efficiency can be controlled by magnetization tuning of a single superconducting island. Moreover, we show that the diode efficiency can be substantially increased in such a device. We argue that the dramatic increase of the diode efficiency is due to competing contributions of the two superconducting islands to the supercurrent with single helical bands linked through the topological insulator surface.

## Introduction

Superconducting nonreciprocal phenomena have been attracting a lot of attention over the last several years [1]. Particularly, the diode effect in superconducting systems has been widely discussed due to its interesting underlying physics and potential application in nondissipative superconducting electronics [2–4]. So far, the superconducting diode effect has been reported in many different systems, including Josephson junctions [5–11], junction-free devices [12–17], superconducting microbridges [18–19], and other systems [20–21]. There have been numerous theoretical propositions demonstrating the possibility of the superconducting diode effect such as bulk superconducting materials [22–34], proximity-effect hybrid structures [35–45], Josephson structures [46–65], nanotubes [66], confined systems [67], asymmetric SQUIDs [68–70], and superconducting systems with nonuniform magnetization [71]. The diode effect might be useful not only from an application point of view, but it may be also employed as a way to detect the spin–orbital coupling (SOC) type of the material [72].

Typically, such devices require three ingredients for achieving the nonreciprocity of the critical current, including lack of inversion and time-reversal symmetries and the presence of the superconducting order parameter [1]. However, it should be emphasized that the lack of inversion symmetry is the implication of the gyrotropy in the structure of the material that supports nonreciprocal transport [39]. On the microscopic level, the lack of inversion symmetry is expressed by the SOC term. In this regard, systems based on topological insulators (TIs) are interesting since they offer strongest SOC rendering linear spin-polarized dispersion for the surface states [73].

The diode effect in TI-based structures has been reported in Josephson junctions, as well as in hybrid structures. In practice, when producing mesoscopic diode devices, it is reasonable to expect some presence of nonmagnetic impurities in the structures. However, it has been shown previously that the diode efficiency is expected to be low in diffusive TI-based systems [37,50]. Another disadvantage of the TI diffusive diodes is their limited tunability. In these devices, the polarity of the diode cannot be changed without reversing the Zeeman field, although in long ballistic S/TI/S (S denotes a superconductor) Josephson junctions such a situation is possible [52].

**Figure 1:**
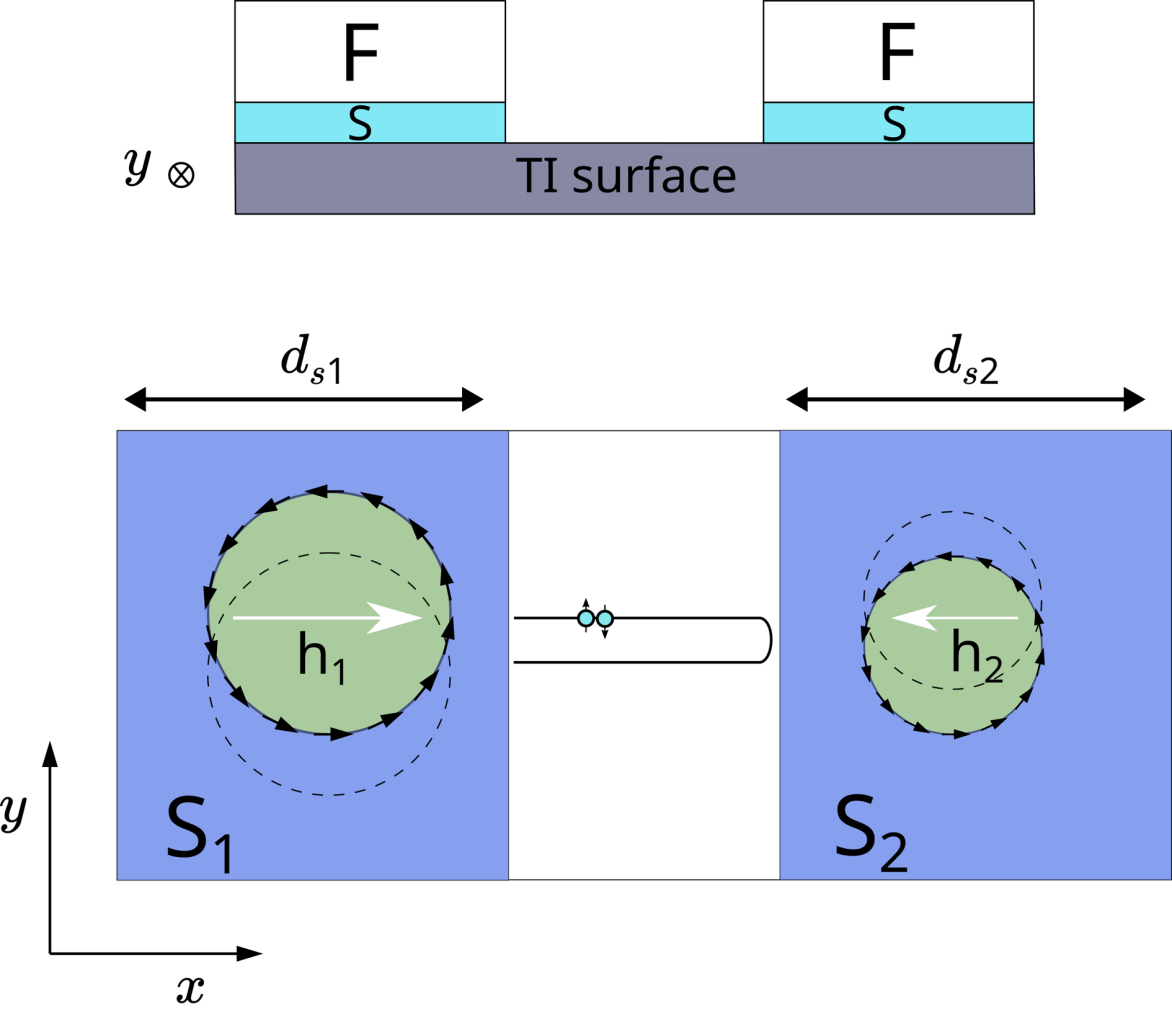
Geometry of the controllable diode under consideration, which consists of two superconducting islands with the proximity-induced in-plane exchange field deposited on top of the topological insulator. Schematic representation of the Fermi contours of the two superconducting regions with the exchange fields oriented in the opposite directions. S_1_ and S_2_ are linked through the TI surface.

In the present work, we propose a superconducting diode based on two superconducting regions with a proximity-induced in-plane exchange field on top of the TI. The Fermi contour of the TI surface states is usually represented by the Dirac spectrum, that is, a single helical band, which is characterized by the strongest spin-momentum locking effect. Here, we consider the F/S/TI/S/F (F denotes ferromagnetic layer) hybrid structure depicted in Figure 1. We argue that such a hybrid structure can behave as a system with two helical bands as, for example, noncentrosymmetric superconductors [26,74]. However, the two helical bands in the structure under consideration are coupled not in the momentum space but in the real space by the TI surface. The coupling between the two islands can be controlled, for example, by the width of the non-superconducting TI part. When considering the diode effect, the proposed layout can substantially increase the diode efficiency, provided the ferromagnetic exchange fields of the two F/S regions are oriented in opposite directions. Misalignment of the exchange fields leads to the competition of the two separate helical bands in the superconducting regions in their contribution to the critical current nonreciprocity (Figure 1).

## Quasiclassical Theory

The F/S/TI/S/F hybrid structure can be described by the following effective low-energy Hamiltonian in the particle–hole and spin space:


[1]

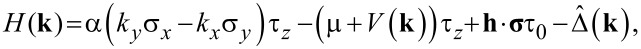



where α is the Fermi velocity, μ is the chemical potential, and *V* is the impurity potential of a Gaussian form, which is used for further quasiclassical approximation in the dirty limit. **h** = (*h_x_*, 0, 0) is the exchange field due to the adjacent ferromagnetic material. The matrices τ and **σ** are 2 × 2 Pauli matrices in the particle–hole and spin spaces, respectively. The superconducting pair potential matrix 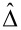 is defined as 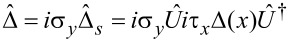, where the transformation matrix is 
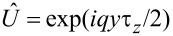
. The finite center of mass momentum *q* takes into account the helical state. The pair potential Δ(*x*) is a real function defined as follows:


[2] $$\Delta \left(x\right)=\{\begin{array}{l} \Delta_{1}(x),\;-d_{s1}-L/2<x<-L/2\\ 0,\;-L/2<x<L/2\\ \Delta_{2}(x),\;L/2<x<d_{s2}+L/2. \end{array}$$

Here, Δ_1_ and Δ_2_ are calculated self-consistently and correspond to the superconducting regions S_1_ and S_2_, respectively (Figure 1). Finally, *L* is the width of the bare TI surface (normal N part) and *d_s_*_1_(*d_s_*_2_) is the width of S_1_ (S_2_) region. It is important to emphasize that, although the geometry of the considered device corresponds to a Josephson junction, in this work we consider zero macroscopic phase difference between regions S_1_ and S_2_, so that the Josephson supercurrent due to the phase shift is absent. The anomalous ground state phase shift ϕ_0_ is also absent since we assume the exchange field component *h_y_* = 0. In contrast, the *h_x_* component is considered to be finite in the system and defined as follows:


[3] $$h_{x}=\{\begin{array}{l} h_{1},\;-d_{s1}-L/2<x<-L/2\\ 0,\;-L/2<x<L/2\\ h_{2},\;L/2<x<d_{s2}+L/2. \end{array}$$

As we stated above, we assume the phase gradient *q* to be the same in the whole system. Obviously, this is not the case if *L* ≫ ξ because, in this case, the Josephson coupling between the S_1_ and S_2_ leads is absent, and they do not “feel” each other. In each lead, a distinct phase gradient *q*_1,2_ = −2*h_i_*/α is established to satisfy the zero spontaneous current condition required for the helical ground state [37,75–76]. If the superconducting leads get closer to each other, Josephson coupling between them develops gradually, and Josephson currents between the leads appear. Consequently, the distribution of the superconducting phase becomes a complex two-dimensional function of spatial coordinates. Thus, a general solution of the problem requires a consideration of the two-dimensional distribution of the order parameter phase; but here we restrict ourselves to the case 
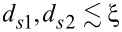
 and the regime of relatively strong Josephson coupling between the leads S_1_ and S_2_. The second condition means that 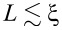, and the transparency of the interfaces between the superconducting leads and the TI layer is rather high. In this case, it is energetically favorable to have the same phase gradient along the whole S_1_/TI/S_2_ Josephson junction and the key results are obtained within this regime.

In practice, one possible implementation of the hybrid structure includes a thin layer of Nb on the surface of Bi_2_Se_3_ with FeMn- or CuNi-based ferromagnets deposited on top of the superconductors. Despite the challenges, it is still possible to implement heterostructures with opposite magnetization directions as in, for example, F/S/F spin valves [77]. Another possibility is more modern and is based on van der Waals structures comprised of transition-metal dichalcogenide materials such as superconducting NbSe_2_ and magnetic VSe_2_ on top of Bi_2_Se_3_ [78].

We solve the stated problem for the Hamiltonian in Equation 1 within the microscopic approach based on the quasiclassical Green’s functions in the diffusive limit, that is, when the coherence length ξ is much larger than the electron mean free path *l*. Such model can be described by the Usadel equations [79–81]


[4]

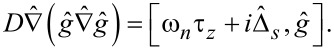



Here *D* is the diffusion constant, and τ*_z_* is the Pauli matrix in the particle–hole space. In the general case, the operator 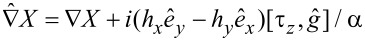. The Green’s function matrix is also transformed as 
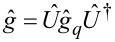
.

To facilitate the solution procedures of the nonlinear Usadel equations, we employ θ parametrization of the Green’s functions [82]:


[5]

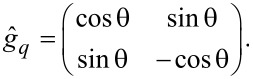



Substituting the above matrix into the Usadel equation (Equation 4), we obtain in the superconducting S parts |x| *> L*/2:


[6]

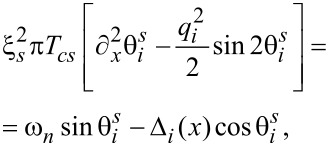



where the indices *i* = 1, 2 refer to the superconducting parts S_1_ and S_2_, respectively, *q_i_* = *q* + 2*h_i_*/α, and in the normal N part −*L*/2 *< x < L*/2:


[7]

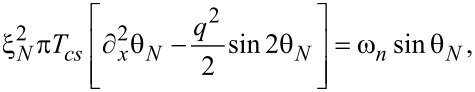



where θ*_s_*_(_*_N_*_)_ means the value of θ is the S(N) of the TI surface, respectively. We introduced the characteristic length 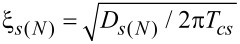, where *D_s_*_(_*_N_*_)_ is the diffusion constant in S(N) part and *T_cs_* is the transition temperature of the bare S region. The self-consistency equations for the pair potentials read


[8]

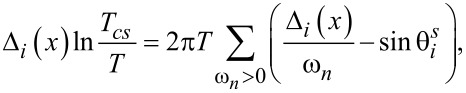



where the summation is performed up to the Debye frequency ω_D_. Finally, we supplement the above equations with two pairs of the boundary conditions (two for each S/N interface) of the following type:


[9]

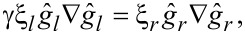




[10]

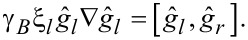



Here, γ_B_ = *R*_B_σ*_l_*/ξ*_l_*, γ = ξ*_r_*σ*_l_*/ξ*_l_*σ*_r_* where σ*_l_*_(_*_r_*_)_ is the conductivity of the material on the left (right) side of the interface. The parameter γ controls the slope of the Green’s functions at the interface, whereas γ_B_ controls the mismatch between the functions at the interface. While for identical materials γ = 1, in general, this parameter may have arbitrary values. γ_B_ is the parameter that determines the transparency of the S/F interface [83–85].

The final problem comprises several equations, namely, the Usadel equations in the superconducting (S) and normal (N) parts (Equation 6 and Equation 7), two self-consistency equations (Equation 8) in each superconducting region S_1_ and S_2_, and the boundary conditions at the S_1_/N, N/S_2_ interfaces and at the free edges of the superconductors. These equations are solved simultaneously for a given phase gradient *q*. Using the finite difference method, the equations are discretized on a one-dimensional grid, resulting in a system of nonlinear equations that is solved by the Newton–Raphson method. We then compute the total supercurrent through the hybrid structure as a function of *q*, from which the supercurrent and critical current of the system are determined.

The supercurrent in the diffusive limit can be found from the expression


[11] $$J_{s(N)}=\frac{-i\pi \sigma_{s(N)}}{4e}T\sum_{\omega_{n}}\text{Tr}\left[\tau_{z}{\hat{g}}_{s(N)}\hat{\nabla }{\hat{g}}_{s(N)}\right].$$

Performing the unitary transformation *U*, the current density transforms as follows:


[12]

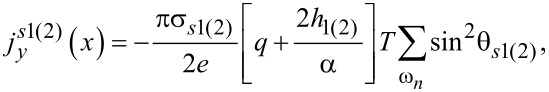




[13]

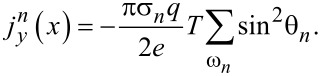



The total supercurrent flowing through the system along the *y*-direction can be calculated by integrating the current density of the total width of the F/S/TI/S/F structure:


[14] $$I=I_{s1}+I_{s2}+I_{\text{N}},$$

where *I_s_*_1_, *I_s_*_2_, and *I*_N_ are the total supercurrents integrated along the *x*-direction in S_1_, S_2_ and N regions, respectively.

## Results and Discussion

**Figure 2:**
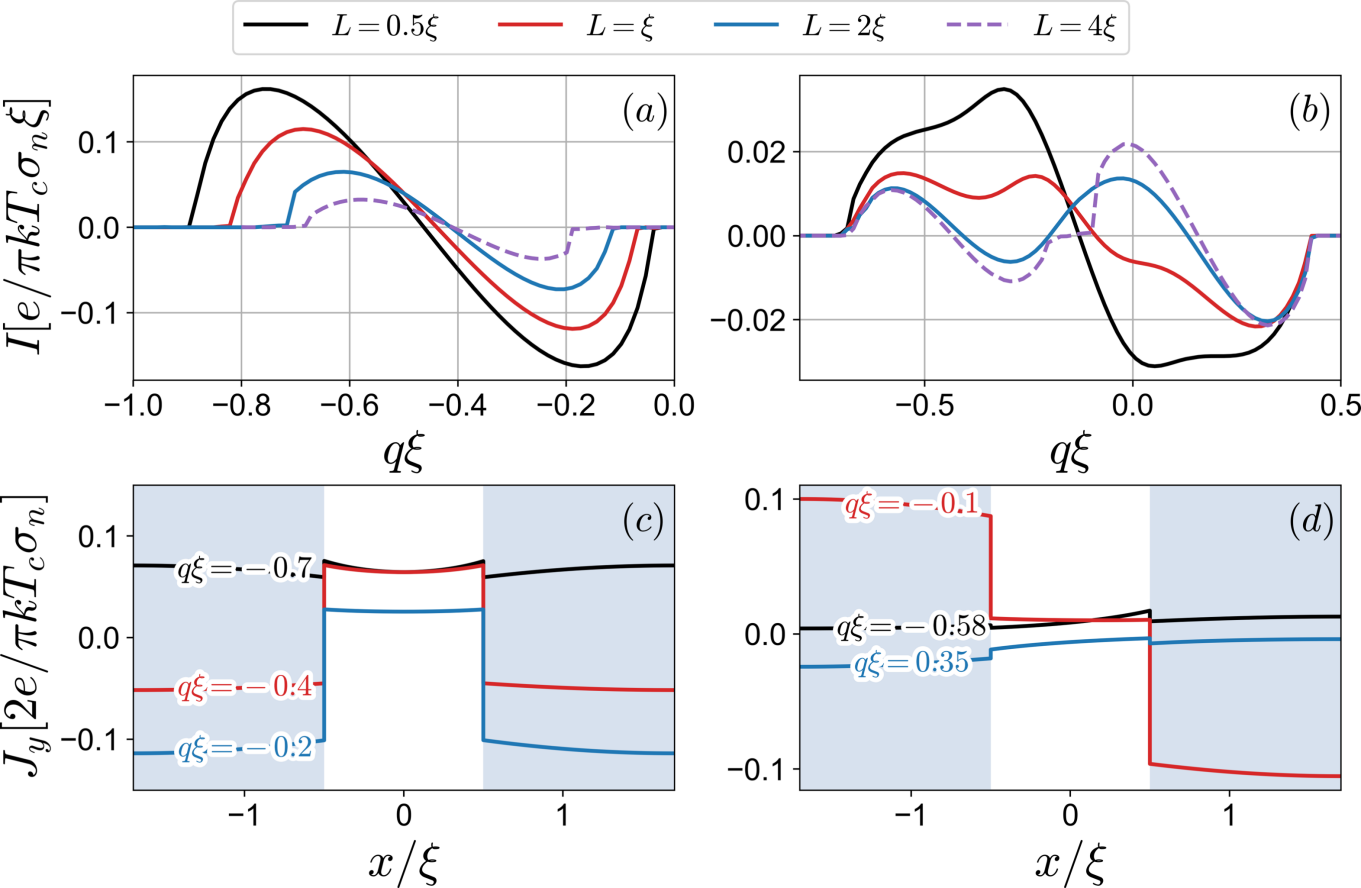
Supercurrent *I* as a function of *q* at *h*_1_ = *h*_2_ = 0.25 (a) and at *h*_1_ = −0.1, *h*_2_ = 0.25 (b). The lower panels illustrate the current density distributions at different *q* corresponding to the upper panels for *L* = ξ.

We fix the following system parameters throughout the discussion of the results: *d_s_*_1_ = *d_s_*_2_ = 1.2ξ, γ_1_ = γ_2_ = 0.5, *T* = 0.1*T_cs_*. We start with the analysis of the *I*(*q*) relations when the exchange fields *H*_1_ and *H*_2_ are the same in both superconducting regions. In Figure 2a, we observe a characteristic behavior of the supercurrent with *I*(*q*_0_) = 0, where *q*_0_ ≠ 0 is the ground-state Cooper pair momentum, which reflects the helical nature of the superconducting ground state. We can also notice some nonreciprocity of the supercurrent, that is, 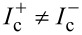, which is a consequence of the helical state. As we will see below, the diode efficiency is quite low and, in this case, does not exceed several percent. In the absence of any exchange field, the supercurrent is *I*(*q* = 0) = 0, which means that the ground state is a conventional state with zero Cooper pair momentum. To get more insight, we plot the supercurrent density *J_y_* in Figure 2b. Hence, in the situation when *H*_1_ and *H*_2_ are perfectly aligned, we expect well-known behavior of the total supercurrent.

Now, we discuss the case when the exchange fields *H*_1_ and *H*_2_ are oriented in opposite directions (Figure 2b). When the distance between S_1_ and S_2_ is large (*L* = 4ξ), the superconducting regions are well separated and act almost independently with distinct critical supercurrents 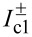 and 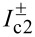 corresponding to S_1_ and S_2_, respectively. This circumstance can be clearly seen from *I*(*q*) dependence for *L* = 4ξ; in this sense, the distance *L* can be imagined as a coupling strength between S_1_ and S_2_. The behavior of *I*(*q*) dramatically changes when *L* becomes smaller. The regions of the *I*(*q*) curve that previously could be easily assigned to each superconducting island start to “overlap”, reflecting stronger coupling between S_1_ and S_2_. As a result, we can achieve a situation in which the critical current of the hybrid structure in one direction is substantially renormalized. For instance, we can observe that 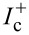 is defined rather by the left maximum of *I*(*q*) at *L* = ξ, while 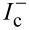 remains approximately at the same value. Stronger coupling between the superconducting regions leads to a more complicated supercurrent density distribution across the hybrid structure (see Figure 2d). Obtaining such nontrivial behavior of *I*(*q*) is the key idea behind achieving a larger diode efficiency η. It should be emphasized that a similar behavior is expected in Rashba superconductors, where the Fermi surface is represented by the two helical bands with the opposite helicities [24–26]. Here, we clearly consider a single-helical-band Fermi surface. However, we can have S_1_ and S_2_ with the opposite *h*_1_ and *h*_2_ in our system as illustrated in Figure 1, which may be thought of as an effective two-helical-bands system.

The diode efficiency can be defined in a standard way, as


[15] $$\eta =\frac{I_{\text{c}}^{+}-\left|I_{\text{c}}^{-}\right|}{I_{\text{c}}^{+}+\left|I_{\text{c}}^{-}\right|}.$$

**Figure 3:**
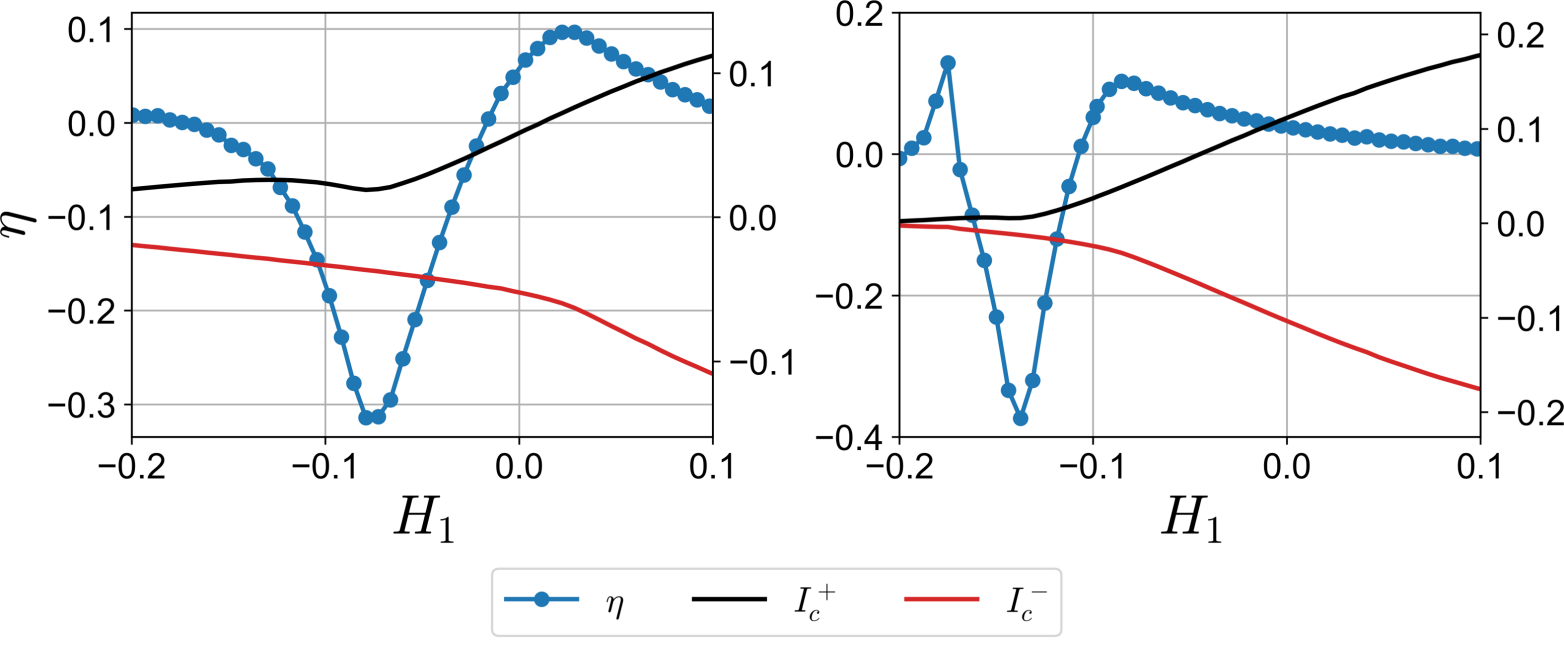
Critical supercurrents 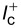 and 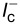 (right vertical scale) and diode efficiency η (left vertical scale) as functions of *H*_1_ for *L* = ξ. Left and right plots correspond to γ_B1_ = γ_B2_ = 0.4 and γ_B1_ = γ_B2_ = 0.2, respectively. The critical currents’ scale is in units of 2*e*/π*kT*_c_σ*_n_*ξ.

**Figure 4:**
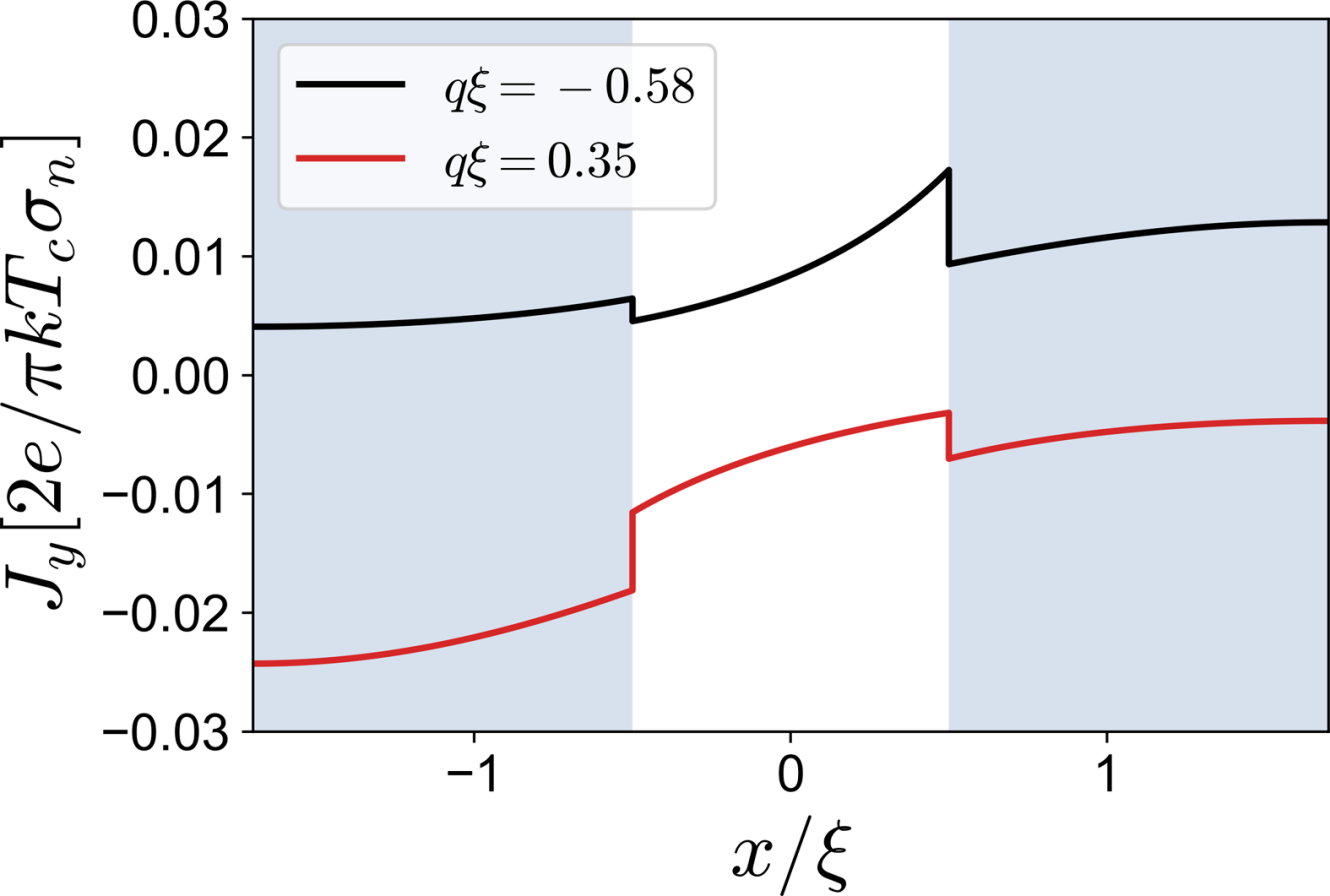
Supercurrent density *J_y_* at *q*ξ = −0.58 (black line) and *q*ξ = 0.35 (red line) calculated at *L* = ξ. All other parameters are the same as in Figure 3.

In Figure 3, the diode efficiency along with the critical currents is demonstrated as a function of *H*_1_, while *H*_2_ is fixed at *H*_2_ = 0.25. We observe several characteristic features of η behavior. First, the diode efficiency is quite low at large positive values of *H*_1_, remaining under 5% at *H*_1_ = 0.1. This is anticipated behavior of the diodes with single helical band in the diffusive limit [37,50,86]. As *H*_1_ decreases, the diode efficiency rises to a certain value, and then η changes its sign rapidly reaching the maximum value. At the point when the diode changes its polarity, there is a transition from S_1_ to S_2_ in their contribution to the critical currents. We assume that in the vicinity of η = 0, the superconducting regions S_1_ and S_2_ strongly compete with each other since, individually, they have opposite efficiencies because *H*_1_ and *H*_2_ are of the opposite signs. We might say that, at a certain value of *H*_1_, the critical currents 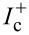 and 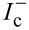 of the total system are predominantly determined by S_1_ and S_2_, that is, the supercurrent mostly passes through one of the superconducting regions in the opposite directions. To better demonstrate this point, we plot the supercurrent density distribution (Figure 4) for values of *q* that correspond to the critical current momenta at *H*_1_ = −0.1, *H*_2_ = 0.25, and *L* = ξ. It can be seen that a larger proportion of the current density is concentrated at the corresponding superconducting region; at *q*ξ = 0.35, *J_y_* is significantly larger at S_1_, while at *q*ξ = −0.58, it is mainly at S_2_. Another important observation from Figure 3 is that the sign change of the diode efficiency occurs at lower values of the critical currents. This means that higher diode efficiencies due to the competition of S_1_ and S_2_ take place in a substantially suppressed superconducting state. Finally, we can see how the interface transparency affects η. Higher transparency can increase the efficiency up to 40%, however at smaller critical currents.

**Figure 5:**
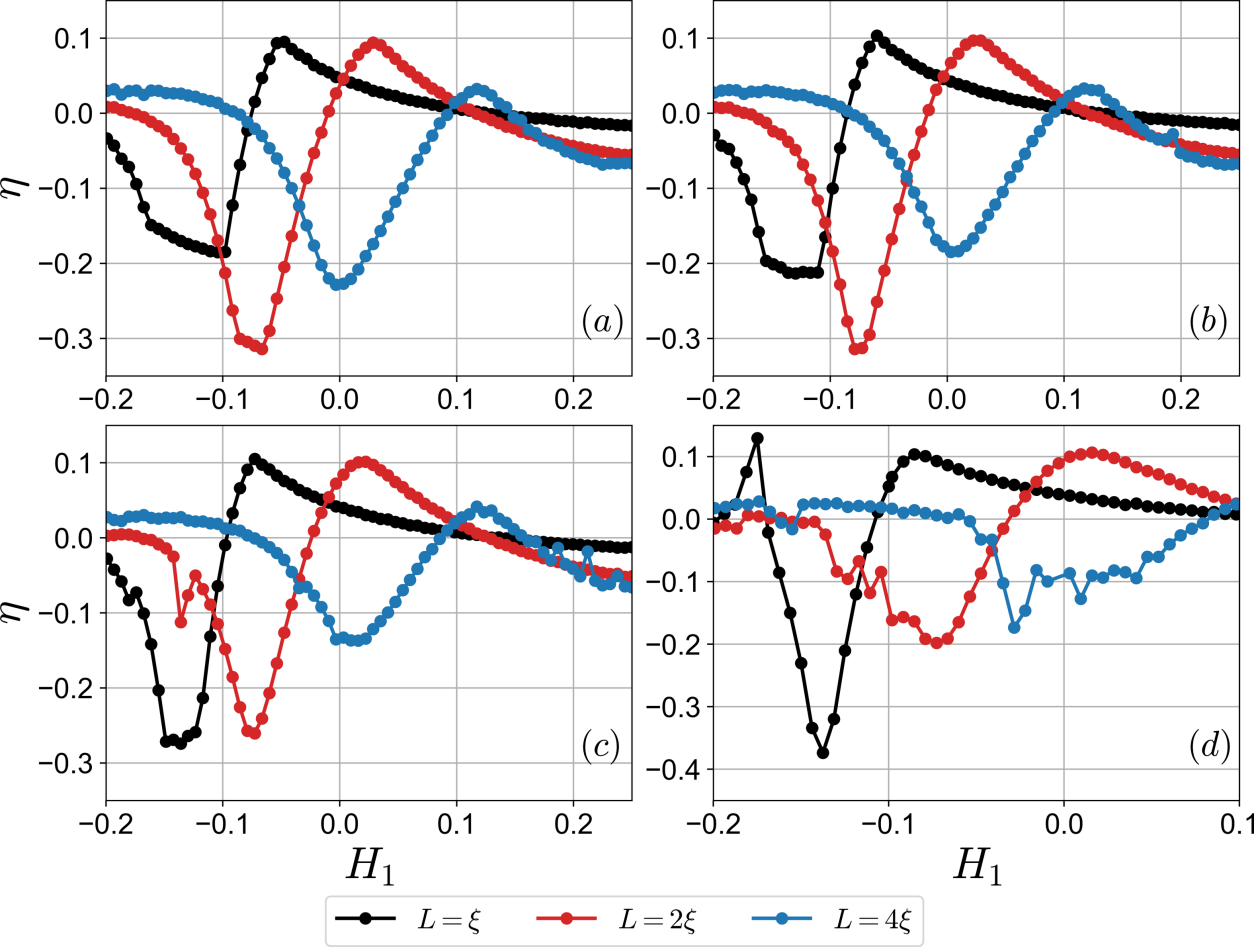
Superconducting diode efficiency η calculated at different interface transparencies γ_B_. Plot (a) corresponds to γ_B1_ = γ_B2_ = 0.5, (b) γ_B1_ = γ_B2_ = 0.4, (c) γ_B1_ = γ_B2_ = 0.3, and (d) γ_B1_ = γ_B2_ = 0.2.

The interface transparency γ_B_ is an important parameter of the system, which, in principle, can be used as a tuning parameter in the experiment. Control of this parameter may be achieved by applying the gating voltage at the interface. We provide more detailed analysis of the interface transparency impact on the diode effect in Figure 5. We notice that the highest efficiency is achieved at smaller γ_B_ = 0.2 for *L* = ξ. However, this is not the general trend as we see from the plots. For instance, the highest η is realized at γ_B_ = 0.5 for *L* = 4ξ. Hence, there exists an optimal value of the interface transparency for the highest efficiency. It is also important to emphasize that the exchange field *H*_1_ at which the “major” sign change of η occurs shifts towards larger values as γ_B_ decreases. This means that the polarity of the diode can be altered via the control of the interface transparency, which cannot be achieved in a diffusive single-helical-band superconducting diode [37]. Finally, we observe repeated sign-changing behavior of the quality factor in Figure 5. This may reflect the competitive nature of the S_1_ and S_2_ behavior in the nonreciprocal supercurrent.

## Conclusion

We have examined the superconducting diode effect in a F/S/TI/S/F hybrid structure. It has been shown that, under the condition that the exchange fields of the ferromagnetic regions are opposite, the diode efficiency can be dramatically increased. Such improvement can be explained in terms of the competitive behavior of the superconducting regions with single helical bands. The obtained results can be useful for achieving highly efficient superconducting diodes in the absence of an external magnetic field. Moreover, the sign of the diode efficiency can be changed as a function of the interface transparency.

As a direction for further studies, one could investigate the Josephson diode effect in the hybrid structure considered in this paper. In this case, the nonreciprocity is achieved in the Josephson critical current.

## Data Availability

Data generated and analyzed during this study is available from the corresponding author upon reasonable request.
